# The Effect of 2-Thiocyanatopyridine Derivative 11026103 on *Burkholderia Cenocepacia*: Resistance Mechanisms and Systemic Impact

**DOI:** 10.3390/antibiotics8040159

**Published:** 2019-09-21

**Authors:** Jaroslav Nunvar, Andrew M. Hogan, Silvia Buroni, Svetlana Savina, Vadim Makarov, Silvia T. Cardona, Pavel Drevinek

**Affiliations:** 1 Department of Medical Microbiology, 2nd Faculty of Medicine, Charles University and Motol University Hospital, V Uvalu 84, 15400 Prague, Czech Republic; pavel.drevinek@lfmotol.cuni.cz; 2 Department of Microbiology, Faculty of Science, University of Manitoba, 213 Buller Building, Winnipeg, MB R3T 2N2, Canada; hogana34@myumanitoba.ca (A.M.H.); silvia.cardona@umanitoba.ca (S.T.C.); 3 Department of Biology and Biotechnology, University of Pavia, Via Ferrata 9, 27100 Pavia, Italy; silvia.buroni@unipv.it; 4 Bach Institute of Biochemistry, Research Center of Biotechnology of the Russian Academy of Sciences, Leninsky Prospect 33, Moscow 119071, Russia; svetl.a.savina@gmail.com (S.S.); makar-cl@ropnet.ru (V.M.); 5 Department of Medical Microbiology and Infectious Diseases, Rady Faculty of Health Sciences, University of Manitoba, 727 McDermot Avenue, Winnipeg, MB R3E 3P5, Canada

**Keywords:** *Burkholderia cenocepacia*, chemical mutagenesis, RNA-Seq, whole-genome sequencing, Tn-Seq, chemical genetics

## Abstract

Bacteria of the *Burkholderia cepacia* complex (Bcc) are associated with significant decline of lung functions in cystic fibrosis patients. Bcc infections are virtually impossible to eradicate due to their irresponsiveness to antibiotics. The 2-thiocyanatopyridine derivative 11026103 is a novel, synthetic compound active against *Burkholderia cenocepacia*. To characterize mechanisms of resistance to 11026103, *B. cenocepacia* was subjected to chemical mutagenesis, followed by whole genome sequencing. Parallel mutations in resistant isolates were localized in a regulatory protein of the efflux system Resistance-Nodulation-Division (RND)-9 (BCAM1948), RNA polymerase sigma factor (BCAL2462) and its cognate putative anti-sigma factor (BCAL2461). Transcriptomic analysis identified positive regulation of a major facilitator superfamily (MFS) efflux system BCAL1510-1512 by BCAL2462. Artificial overexpression of both efflux systems increased resistance to the compound. The effect of 11026103 on *B. cenocepacia* was analyzed by RNA-Seq and a competitive fitness assay utilizing an essential gene knockdown mutant library. 11026103 exerted a pleiotropic effect on transcription including profound downregulation of cluster of orthologous groups (COG) category “Translation, ribosomal structure, and biogenesis”. The competitive fitness assay identified many genes which modulated susceptibility to 11026103. In summary, 11026103 exerts a pleiotropic cellular response in *B. cenocepacia* which can be prevented by efflux system-mediated export.

## 1. Introduction

*Burkholderia cepacia* complex species (Bcc) comprises bacteria from diverse environmental and clinical sources [1]. They typically infect hospitalized patients with underlying medical conditions. Patients with cystic fibrosis (CF) are among the ones at the greatest risk of contracting a Bcc infection. Bcc thriving in thick CF sputum are associated with chronic inflammation and significant decline in lung functions; infections with the most virulent Bcc lineages ultimately result in death due to a necrotizing septic pneumonia (cepacia syndrome) [2,3].

Poor prognosis of Bcc infections in CF patients is largely a consequence of the irresponsiveness of the microbes to antibiotic treatment. Bcc are intrinsically resistant to several antibiotic classes [4]. In addition, the large pulmonary Bcc populations present at chronic infections rapidly evolve acquired resistance to virtually all clinically available antibiotics [5,6], making the infections impossible to be eradicated by antibiotic therapy. Thus, there is an urgent need to develop novel antibacterial therapies to combat Bcc infections in CF.

The compound 11026103 is one of the recently discovered, synthetic 2-thiocyanatopyridine derivatives which were originally found to have antibacterial activity against *Mycobacterium tuberculosis* [7]. Subsequent work demonstrated that 11026103 also inhibited growth of *Burkholderia cenocepacia* [8]. This study aimed to characterize the mechanisms of resistance of *B. cenocepacia* to 11026103 and to elucidate the antibacterial mechanism of action.

## 2. Results and Discussion

### 2.1. Selection and Genetic Characterization of B. cenocepacia Mutants Resistant to 11026103

Previous work [8] showed that resistance to 11026103 in spontaneously arising mutants of *B. cenocepacia* J2315 was exclusively due to overexpression of the efflux system Resistance-Nodulation-Division (RND)-4 [9]. To uncover additional mechanisms conferring resistance to 11026103, which could point to a molecular target of this compound, we performed chemical mutagenesis. Two strong mutagens with different spectra of induced mutations were used: ethyl methanesulfonate (EMS), which induces GC→AT transitions, and 5-azacytidine (5-azaC), which induces mostly GC→CG transversions in *Escherichia coli* [10]. The deletion derivative of *B. cenocepacia* J2315 which lacked RND-4 [9] was chosen as a parental strain for experiments to prevent selection of mutants overexpressing this efflux system [8].

**Figure 1:**
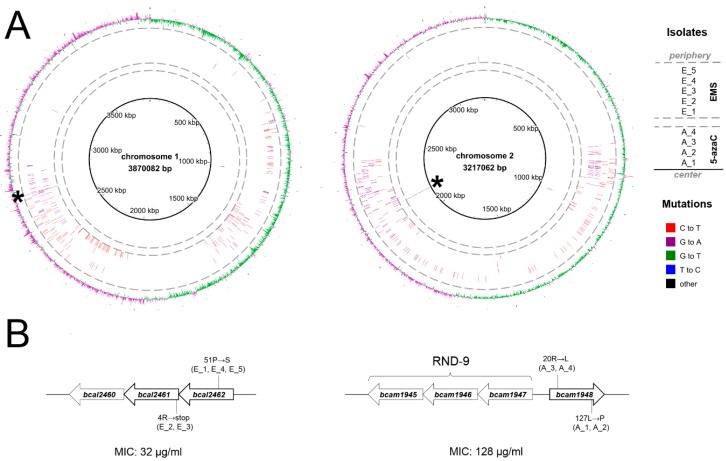
Mutations in *B. cenocepacia* isolates resistant to 11026103. (**A**) All mutations, mapped to chromosomes 1 and 2, which resulted from chemical mutagenesis with ethyl methanesulfonate (EMS) (five isolates; outer circles) or 5-azaC (four isolates; inner circles). The genes/operons which displayed convergent mutations are denoted by asterisks. (**B**) Detailed view of parallel mutations. MICs of corresponding 11026103-resistant mutants are denoted. The visualizations were carried out using the BRIG software [12].

Five and four mutants resistant to 11026103 were obtained using EMS and 5-azaC mutagenesis, respectively (see Materials and Methods): E_1–E_5 and A_1–A_4 (Figure 1). To identify mutations which resulted from mutagenic treatments, whole-genome sequencing (WGS) of all resistant isolates was conducted and the obtained genomic sequences were compared to the reference genome of *B. cenocepacia* J2315 (see Materials and Methods). The amount and spectrum of mutations induced by each mutagen differed profoundly. 5-azaC mutagenesis produced only single mutations, which in all mutants localized to the *bcam1948* gene (Figure 1), encoding a transcriptional regulator of the RND-9 efflux system [11]. The resistant mutants A_1 and A_2 both harbored an identical 127L→P mutation; the resistant mutants A_3 and A_4 both harbored an identical 20R→L mutation in BCAM1948. Arginine 20R is a conserved residue in BCAM1948 homologs whose mutations lead to overexpression of the RND-9 efflux operon [11]. These results thus suggest that overexpression of RND-9 could increase resistance to 11026103.

Virtually all EMS-induced mutations were C→T or G→A transitions, which is consistent with the known mutagenic effect of EMS [10,13]. EMS-induced transitions occurred as long stretches (>100 kbp) composed exclusively of one type of transition. Furthermore, on both chromosomes 1 and 2, mutated regions showed roughly symmetrical distribution around the replication terminus (Figure 1a). These stretches of mutations are likely to have originated from segments of replicating DNA which escaped repair mechanisms; the symmetrical distribution between chromosomes 1 and 2 reflects coordinated replication of both replicons [14]. The only genetic locus where mutations converged among all isolates selected after EMS mutagenesis was the *bcal2461-bcal2462* gene pair (Figure 1b). Three isolates harbored an identical mutation in BCAL2462 (51P→S). This gene encodes an alternative sigma factor 70 of RNA polymerase [15]. The remaining two isolates harbored an identical mutation in BCAL2461 (4R→stop), which introduces a premature stop codon. *bcal2461* lacks functional annotation; however, its physical linkage with the sigma factor gene *bcal2462* implies its function as an anti-sigma factor, as these two gene categories typically co-occur in operons.

### 2.2. Resistance of B. cenocepacia to 11026103 Is Solely Efflux-Mediated

**Figure 2:**
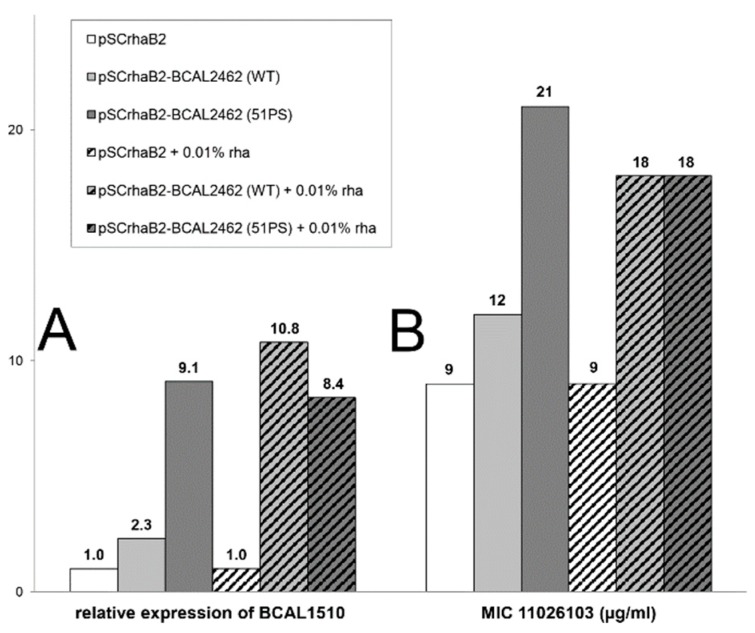
The effect of artificial expression of BCAL2462 (wild-type (WT) or 51P→S allele) in *B. cenocepacia* J2315 on (**A**) expression of major facilitator superfamily (MFS) transporter BCAL1510; (**B**) MIC of 11026103. Expression changes were calculated in comparison with empty vector control experiment (J2315 + pSCrhaB2 with or without rhamnose).

Since sigma factors are transcriptional activators of genes involved in various cellular processes, we hypothesized that the association of mutations in BCAL2462 or BCAL2461 with resistance to 11026103 was indirect. To clarify which genes were transcriptionally activated by BCAL2462, RNA-Seq analysis was conducted. Both the wild-type (WT) allele and the 51P→S allele of the sigma factor BCAL2462 were cloned into expression vector pSCrhaB2 and transformed into *B. cenocepacia* J2315. The effect on gene expression was assessed for both alleles with and without the addition of inducer. The greatest extent of transcriptional induction was observed for the entire *bcal2460-bcal2462* operon, in accordance with autoregulation of sigma-factor-encoding loci being common [16]. In addition, the expression of the entire operon *bcal1510-bcal1512*, which encodes a major facilitator superfamily (MFS) efflux system, was largely induced by BCAL2462 (Figure S1). This induction depended on the sigma factor allele. WT BCAL2462 expressed from pSCrhaB2 required induction by rhamnose to fully activate expression of *bcal1510*. In contrast, the 51P→S allele, which was associated with resistance to 11026103, caused full induction of *bcal1510* even in the absence of rhamnose (Figure 2a), likely due to some leakiness of the rhamnose-inducible promoter of multicopy plasmid pSCrhaB2. This demonstrates that the sigma factor BCAL2462 activates expression of the MFS efflux system BCAL1510-12 and that this activation is increased in the 51P→S mutant. To further investigate the link between BCAL2462, BCAL1510-12, and resistance to 11026103, MICs of 11026103 were determined under the same conditions which were used in the transcriptomic experiments. The MICs correlated with expression levels of the efflux system; the 51P→S allele of BCAL2462 conferred resistance even in the absence of induction (Figure 2b). These observations imply that overexpression of the MFS efflux pump BCAL1510-12 is a mechanism of resistance to 11026103.

**Figure 3:**
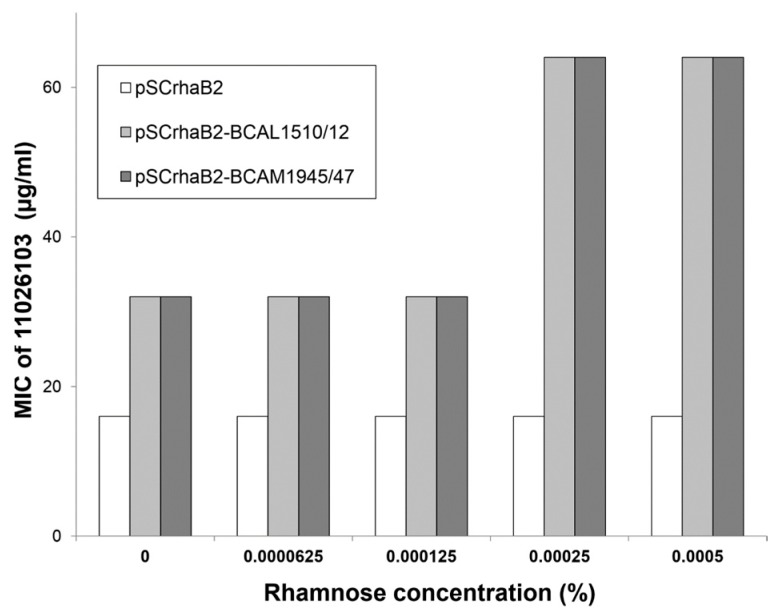
The effect of artificial expression of efflux systems BCAL1510-1512 (MFS) and BCAM1945-1947 (RND-9) in *B. cenocepacia* J2315 on MIC of 11026103. MICs were determined in media containing increasing concentrations of expression inducer (L-rhamnose) up to 0.0005% (*w*/*v*). Rhamnose concentrations of 0.001% (*w*/*v*) and higher impaired viability of bacteria overexpressing both efflux systems, regardless of the presence of 11026103.

All mutations inferred from WGS to be causal of resistance to 11026103 were located in transcriptional regulators of efflux pumps. To definitively prove the ability of both transporters to protect *B. cenocepacia* from the detrimental effect of 11026103, both efflux system operons (*bcal1510-1512* and *bcam1945-1947*) were cloned into pSCrhaB2 and transformed into *B. cenocepacia* J2315. For the resulting transformants, MICs of 11026103 were determined at different concentrations of expression inducer (Figure 3). Even in the absence of induction, bacteria expressing either one of the efflux systems had their MIC elevated twice in comparison with an empty vector control, suggesting that the background (leaky) expression provided significant protection against 11026103. At higher inducer concentrations, the MICs further increased up to four-fold over control values. Higher resistance levels could not be observed because at rhamnose concentrations ≥0.001% (*w*/*v*), growth of bacteria was impaired regardless of the presence or concentration of 11026103. We thus conclude that both efflux systems actively expel 11026103 from *B. cenocepacia* cells, thereby conferring resistance.

In line with the previous attempt to characterize resistance of *B. cenocepacia* to 11026103 [8], our mutagenic approach identified only efflux-based resistance mechanisms. No mutations in essential proteins were associated with resistance to 11026103, in contrast with protein-targeting antibiotics, where resistance arises by target site mutations. As chemical mutagenesis greatly increases the frequency of target site mutations [17], this suggests that the antibacterial action of 11026103 proceeds via a different mechanism than binding to a conserved proteinaceous target.

### 2.3. Knockdowns in Transport and Metabolic Genes Increase Resistance to 11026103

**Figure 4:**
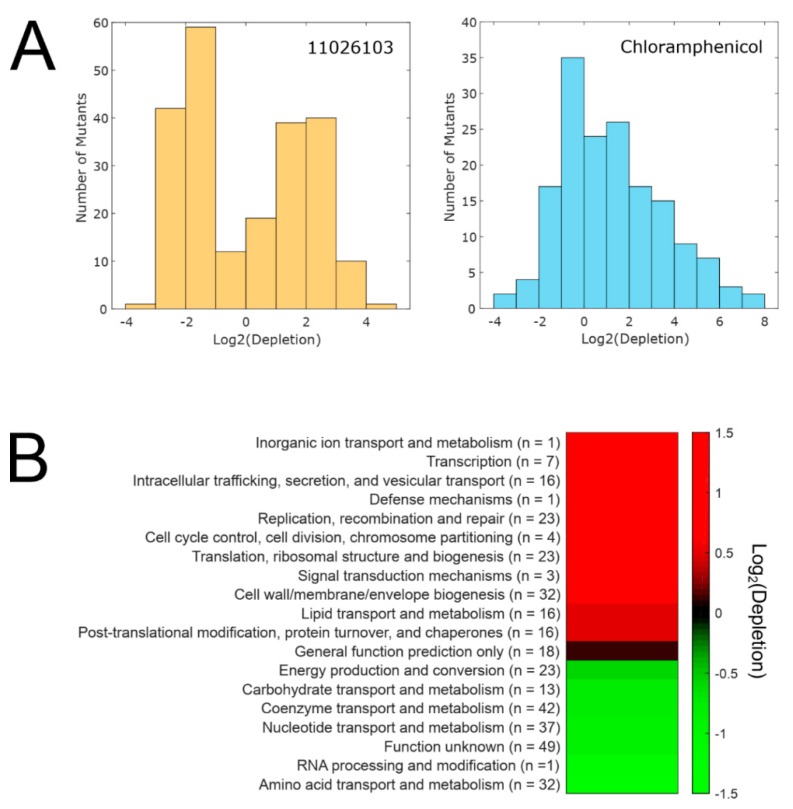
Effect of 11026103 on essential gene knockdown mutants. (**A**) The bimodal distribution of genes affected by 11026103. Normal distribution, resulting from chloramphenicol treatment, is shown for comparison. Abundance of each mutant relative to the no antibiotic control was calculated by taking the log_2_ of the normalized read count for the control divided by the normalized read count for the 11026103- or chloramphenicol-treated sample. Positive values indicate susceptibility. (**B**) Cluster of orthologous groups (COG) categories affected by 11026103. The values for individual genes per COG category were averaged. The total number of genes recovered after the growth with 11026103 is shown for each COG category.

Since the mutagenic protocol did not yield any target site mutants, we employed an alternative approach to investigate the mechanism of action of 11026103, utilizing a library of essential gene knockdown mutants in *B. cenocepacia* K56-2 [18]. Each mutant has an integrated rhamnose-inducible promoter, allowing tunable control of downstream gene expression [19]; when antibiotic targets or resistance determinants are knocked down, the now susceptible cells are depleted from the growth pool. For antibiotics with a single protein target molecule, the distribution of depletions appears approximately normal, with most mutants showing no changes in relative abundance, and with several highly depleted mutants which are candidates for under-expressing the antibiotic target [18]. In contrast, when the mutant library was exposed to the subinhibitory concentration of 11026103 (a concentration that inhibited 25% of growth (IC_25_), see Materials and Methods), the distribution of mutants appeared bimodal (Figure 4a). Many mutants in genes from diverse cluster of orthologous groups (COG) categories were hypersusceptible to 11026103, while mutants in other COG categories displayed increased resistance to the compound (Figure 4b). Importantly, knockdown of genes involved in transcription, intracellular trafficking, replication, translation, and cell wall biogenesis caused hyper-susceptibility to 11026103. Genetic networks link seemingly distant cellular processes [20,21]; therefore, if 11026103 were to interfere with one or more nodes in an essential network, one would expect that diverse processes would be affected.

Mutants with knockdown in biosynthetic genes for the metabolism and transport of carbohydrates, coenzymes, nucleotides, and amino acids were more abundant after exposure to 11026103, indicating they were less susceptible. From a previous report on the activity of a structurally related 2-thiopyridine compound [22] against *M. tuberculosis* [23], unknown intracellular enzymes were found to be necessary in converting these compounds to their active forms. It therefore remains possible that 11026103 is also modified by intracellular enzymes to become active, and that knocking down these biosynthetic enzymes in our assay reduced the conversion of 11026103, thereby conferring a small amount of protection to the cells. Alternatively, knockdown of these metabolic processes could have slowed the growth rate, again conferring a small protective effect.

### 2.4. Transcriptomic Response of B. cenocepacia Elicited by 11026103

The mechanism of antibacterial action of HTP-2b, a structurally related 2-thiopyridine compound, was recently determined. HTP-2b shuttles divalent copper ions from the culture medium into the bacterial cytoplasm, resulting in copper poisoning [23]. Although related, 11026103 and HTP-2b display several differences in their chemical structure (Figure S2). We thus tested if divalent cations of copper (or other transition metals, namely, manganese, cobalt, zinc, and nickel) could potentiate the antibacterial effect of 11026103. None of the metals increased MICs of 11026103 against *B. cenocepacia* J2315 (Table S3), suggesting a different or more complex mode of action.

To characterize the antibacterial mechanism of 11026103, a transcriptomic approach was chosen. *B. cenocepacia* J2315 was challenged with increasing concentrations of 11026103 (ranging from sub-inhibitory to inhibitory concentrations; Figure S3) and changes in transcription of individual genes were determined by RNA-Seq. Three arbitrary thresholds of fold change of expression (≥3, ≥5, and ≥10) were applied in order to select the genes whose expression was most affected by 11026103. These genes and pathways were assumed to point to the molecular processes involved in the growth-inhibitory effect of 11026103.

**Figure 5:**
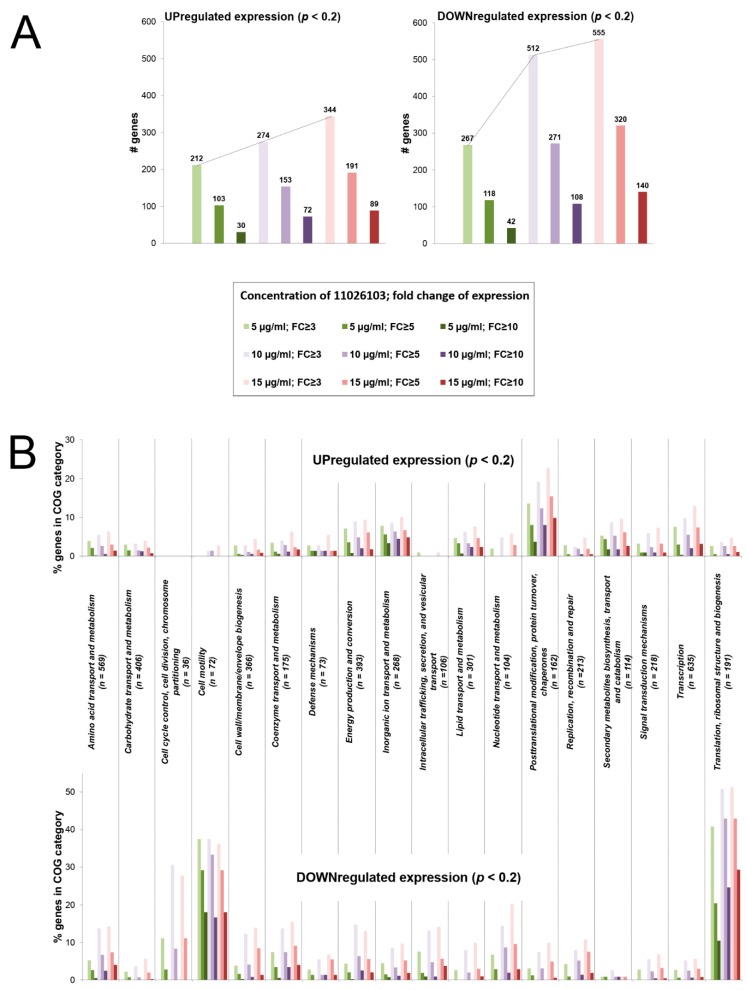
Effect of 11026103 on transcriptome of *B. cenocepacia* J2315. The differentially expressed genes are color-coded according to the concentration of 11026103 used (sub-inhibitory: 5 µg mL^−1^; inhibitory: 10 µg mL^−1^ and 15 µg mL^−1^). The extent of differential expression (fold-change (FC) of expression with respect to untreated control experiment) is denoted by the intensity of corresponding colors. Genes whose differential expression did not reach statistical significance (*p* > 0.2) were discarded from the calculations. (**A**) Total numbers of differentially expressed genes. (**B**) Representation of COG categories among differentially expressed genes. Total numbers of *B. cenocepacia* J2315 genes in each COG category are denoted.

11026103 exerted a profound effect on transcription (Figure 5), suggesting an extensive systemic impact on bacterial cells. At all concentrations of 11026103 used, the overall number of downregulated genes was greater than the number of upregulated genes (Figure 5a). For example, using the highest concentration of 11026103 (15 µg mL^−1^), the transcription of 344 and 555 genes (4.7% and 7.6% of total genes) was upregulated and downregulated, respectively, more than three-fold. The total number of upregulated genes increased linearly with increasing concentrations of 11026103. In contrast, the total number of downregulated genes increased sharply (approximately doubled) between sub-inhibitory and inhibitory concentrations of the compound (Figure 5a). The growth-inhibitory effect of 11026103 is thus accompanied by a generalized downregulation of transcription.

To identify which cellular processes were affected by 11026103, the differential expression data were compared among COG categories (Figure 5b). For genes upregulated by 11026103, the most affected COG category was “Posttranslational modification, protein turnover, and chaperones”. The COG categories which showed the most profound downregulation by 11026103 were “Cell motility” and “Translation, ribosomal structure, and biogenesis” (Figure 5b). Motility shutdown might reflect the burden of this nonessential, energetically costly pathway [24] under conditions imposed by 11026103. The downregulation of translation apparatus is arguably the most serious consequence of exposure to 11026103, as it involves almost a half of total genes in this category (*n* = 191) (Figure 5b). Transcriptional downregulation of translation machinery is not commonly observed in response to antibiotics [25]. Detailed analysis of translation-associated genes shows far greater repression at inhibitory concentrations of 11026103 than at subinhibitory concentrations. Furthermore, all components of the translational apparatus (ribosomal proteins, translation factors, RNA processing/modification, and amino acyl-tRNA synthases) were represented among the genes highly repressed by 11026103 (Figure S4). This extent of repression suggests that 11026103 directly or indirectly affects global regulatory circuits, e.g., the stringent response, the major bacterial pathway of translation shutdown [26]. Altogether, the growth-inhibitory effect of 11026103 against *B. cenocepacia* is likely to result from a halt of proteosynthesis.

## 3. Materials and Methods

### 3.1. Bacterial Strains, Plasmids, and Growth Conditions

The bacterial strains and plasmids used in this work are listed in Table S1. Luria-Bertani (LB) broth or LB agar (Sigma Aldrich, St. Louis, MO, USA) were used for all cultivations. Cultures were routinely grown aerobically at 37 °C in static (thermostat) or shaken (150 rpm) environment. Cultivation media were supplemented with the following sterile stock solutions of antimicrobials to desired concentrations: trimethoprim (50 mg mL^−1^ in DMSO), kanamycin (40 mg mL^−1^ in water), ampicillin (50 mg mL^−1^ in water), and 11026103 (10 mg mL^−1^ in DMSO).

### 3.2. Chemical Mutagenesis

The mutagenesis protocol was modified from [10]. For EMS mutagenesis, cells from 10 mL log-phase culture of *B. cenocepacia* D4 [9] were harvested by centrifugation and resuspended in 10 mL of sterile PBS. Three hundred and fifty microliters of EMS (Sigma Aldrich) was added and the mixture was incubated aerobically at 37 °C for 45 min. Cells were harvested by centrifugation, resuspended in 10 mL of LB and cultivated for another 3 h. For 5-azaC mutagenesis, 1 µL of log-phase culture of *B. cenocepacia* D4 was inoculated into 1 mL of LB supplemented with 5-azaC (100 µg mL^−1^) and incubated aerobically at 37 °C overnight. After mutagenesis, selection of 11026103-resistant mutants was performed on LB agar plates containing 50 µg mL^−1^ of 11026103 for 3 days. Suspected resistant mutants were streaked onto LB/11026103 plates and a single isolated colony was frozen at −80 °C.

### 3.3. MIC Determination

Bacterial growth from an overnight agar plate was transferred into 1 mL Mueller-Hinton (MH; Sigma Aldrich) to obtain a suspension with optical density (OD_600_) approximately 0.05–0.1. The cultures were incubated at 37 °C with shaking for 2 h to reach a mid-exponential phase. A sterile solution of 11026103 in MH was diluted with sterile MH to obtain a concentration gradient. One hundred microliters of 11026103 solutions were transferred to MIC microtiter plate wells and inoculated with 1 µL of bacterial cultures. The MIC plates were incubated aerobically for 24 h at 37 °C and MICs were recorded as the minimal concentration of antimicrobial compound which resulted in no visible growth. The experiments were repeated in three biological replicates.

### 3.4. Construction of pSCrhaB2 Derivatives and Genetic Modification of B. cenocepacia

For PCR amplification of the genes of interest, DNA isolated with ChargeSwitch gDNA Mini Bacteria Kit (Invitrogen, Waltham, MA, USA) was used as template. Q5 Hot Start High-Fidelity DNA Polymerase (New England Biolabs, Ipswich, MA, USA) was used for amplification according to the manufacturer’s instructions; the primers are listed in Table S2. The gel-purified PCR products (GeneJET Gel Extraction Kit, Thermo Scientific, Waltham, MA, USA) and plasmid pSCrhaB2 [27] were double-digested with NdeI and HindIII (New England Biolabs). After ligation (Quick Ligation Kit, New England Biolabs), the mixtures were transformed into Library Efficiency DH5α™ Competent Cells (Invitrogen) and transformants were selected on LB plates supplemented with trimethoprim (50 µg mL^−1^). Plasmids were extracted using ChargeSwitch-Pro Plasmid Miniprep Kit (Invitrogen) and the correct insert size was confirmed following NdeI/HindIII double-digestion by gel electrophoresis. Plasmids were introduced into *B. cenocepacia* J2315 by triparental mating as described [27]; transconjugants were selected on LB plates supplemented with trimethoprim (200 µg mL^−1^).

### 3.5. Whole-Genome Sequencing and Data Analysis

Preparation of sequencing libraries and whole-genome sequencing were performed as described in [3]. Sequencing reads were mapped to the complete reference genome of *B. cenocepacia* J2315 [28] using Geneious R9 platform [29]. The in-house Geneious read mapper was used with the following custom mapping settings: maximum 20% gaps per read, maximum 10% mismatches per read. Variants with frequency ≥80% and coverage ≥10 were called using the Geneious in-house functionality. Single-nucleotide polymorphisms (SNPs) with average quality lower than 25 and SNPs present in genomes of all 11026103-resistant mutants (false positive mutations) were discarded. Sequencing reads are available from GenBank (Bioproject PRJNA484695).

### 3.6. RNA-Seq and Data Analysis

To investigate the transcriptomic effect of BCAL2462 expression, 10 mL cultures of *B. cenocepacia* J2315 carrying pSCrhaB2, pSCrhaB2-BCAL2462(WT), or pSCrhaB2-BCAL2462(51PS) were grown to mid-log phase and L-rhamnose was added to a final concentration of 0.01% (*w*/*v*). To parallel cultures, an equal volume of water was added. To investigate the transcriptomic effect of 11026103, 10 mL cultures of *B. cenocepacia* J2315 were grown to mid-log phase and 11026103 was added to sub-inhibitory (5 µg mL^−1^) or inhibitory (10 µg mL^−1^ or 15 µg mL^−1^) final concentrations. To the control (mock) culture, an equal volume of DMSO was added. Cultures were incubated for 20 min after the addition of 11026103, rhamnose, or mock treatment and rapidly cooled in an ice/water bath, and cells were harvested by centrifugation (5000× *g*, 10 min, 4 °C). RNA was isolated using the RNAqueous Total RNA Isolation Kit (Invitrogen) and treated with TURBO DNA-*free* Kit (Invitrogen). cDNA sequencing libraries were prepared using the ScriptSeq Complete Kit (Illumina, San Diego, CA, USA) and sequenced on the MiSeq platform (Illumina) using MiSeq Reagent Kit v2 (300 cycle) (Illumina); sequencing reads are available from Genbank (Bioprojects PRJNA484696 and PRJNA484697). Sequencing reads were mapped to the complete reference genome *B. cenocepacia* J2315 [28] and expression levels were calculated for all annotated genes. Normalization of expression levels between samples (Median of Gene Expression Ratios method), calculation of differential expression level, and statistical testing of differential expression were all conducted using the in-house functionalities in Geneious R9 [29]. Genes were assigned to COG categories according to www.burkholderia.com [30].

### 3.7. Competitive Fitness Assay and Data Analysis

Experiments were performed as previously reported [19] with minor modifications. Briefly, pools of approximately 1000 knockdown transposon mutants of *B. cenocepacia* K56-2 were inoculated in 5 mL LB tubes at a starting OD_600_ of 0.0025. L-rhamnose was added to 0.02% and 0.04% (*w*/*v*), enough for the cultures to reach approximately 30–60% of wild-type K56-2 growth. 11026103 was added to a concentration that inhibited 25% of growth (IC_25_) of the mutant pools (0.7 µg mL^−1^ for 0.02% rhamnose and 0.9 µg mL^−1^ for 0.04% rhamnose). In parallel, the mutant pools were grown in the presence of chloramphenicol (IC_25_: 5 µg mL^−1^). Control mutant pools were grown in the absence of antibacterial agents. Cells were harvested after 20 h of shaking at 37 °C and gDNA was extracted and processed for Tn-Seq as described previously [19]. The Illumina MiSeq platform at Génome Québec was used for sequencing Tn-genome junctions. Raw sequencing reads are available from Genbank (Bioproject PRJNA494611). Data was processed as previously described [19] using custom scripts available from https://github.com/mdomarat/CardonaLab, except that a threshold of 10 reads was applied to insertion sites in the 11026103-free controls to remove sequencing artifacts (e.g., insertion sites known to be absent in the starting pool).

## 4. Conclusions

The whole body of data obtained in this work suggests that the antibacterial effect of 11026103 on *B. cenocepacia* is mediated via a general (systemic) mechanism. The transcriptomic response elicited by 11026103 affected large numbers of genes and processes and a similar situation was observed in our competitive fitness assay of essential gene knockdown mutants. The exact mechanism of action of 11026103 thus remains unknown; the transcriptomic results imply repression of translation at the core of its growth-inhibitory effect. 11026103 is exported by both RND-4 [8] and RND-9; these efflux systems provide wide-range protection not only from several classes of classical antibiotics [9,31,32] but also from disinfectants [33,34], natural compounds [35], and novel drugs [11]. Altogether, this stresses the utility of RND efflux pump inhibitors [36] in increasing susceptibility of Bcc to antimicrobials and in the prevention of development of high-level resistance to both established and novel therapeutic drugs.

## Supplementary Materials

The following are available online at https://www.mdpi.com/2079-6382/8/4/159/s1. Figure S1: The effect of BCAL2462 expression on the transcriptome of *B. cenocepacia* J2315, Figure S2: Comparison of chemical structure of 11026103 and HTP-2b, Figure S3: Concentration-dependent inhibitory effect of 11026103 on *B. cenocepacia* J2315, Figure S4: Effect of 11026103 on transcription of COG “Translation, ribosomal structure and biogenesis”, Table S1: Bacterial strains and plasmids used in this work, Table S2: Primers used for PCR amplification, Table S3: MIC of 11026103 against *B. cenocepacia* J2315 in the presence of transition metals.
