# Supplementary material for: The Effect of 2-Thiocyanatopyridine Derivative 11026103 on Burkholderia Cenocepacia: Resistance Mechanisms and Systemic Impact
Source: Antibiotics (Basel). 2019 Sep 21;8(4):159. doi: 10.3390/antibiotics8040159 (PMC6963507; doi:10.3390/antibiotics8040159)

Table S1. Bacterial strains and plasmids used in this work.

Strains	Genotype	Phenotype	Source
B. cenocepacia J2315	WT	WT	lab stock
B. cenocepacia K56-2	WT	WT	lab stock
B. cenocepacia D4	J2315 Δ*bcal2820–bcal2822*	multidrug resistance (RND-4 efflux system deletion)	(1)
B. cenocepacia JN1	J2315 pSCrhaB2-BCAL2462 (WT)	conditional expression of sigma factor BCAL2462 (WT)	this work
B. cenocepacia JN2	J2315 pSCrhaB2-BCAL2462 (51PS)	conditional expression of sigma factor BCAL2462 (51PS)	this work
B. cenocepacia JN3	J2315 pSCrhaB2-BCAL1510/12	conditional expression of MFS efflux system BCAL1510-1512	this work
B. cenocepacia JN4	J2315 pSCrhaB2-BCAM1945/47	conditional expression of RND-9 efflux system	this work
**Plasmids**			
pSCrhaB2	ori_{pBBR1}, *rhaR*, *rhaS*, P_{rhaB}, Tp^{r}, mob^{+}		(2)
pSCrhaB2-BCAL2462 (WT)	pSCrhaB P_{rhaB}::*bcal2462(WT)*		this work
pSCrhaB2-BCAL2462 (51PS)	pSCrhaB P_{rhaB}::*bcal2462(51PS)*		this work
pSCrhaB2-BCAL1510/12	pSCrhaB P_{rhaB}::*bcal1510-1512*		this work
pSCrhaB2-BCAM1945/47	pSCrhaB P_{rhaB}::*bcam1945-1947*		this work

1. Buroni S, Pasca MR, Flannagan RS, Bazzini S, Milano A, Bertani I, et al. Assessment of three Resistance-Nodulation-Cell Division drug efflux transporters of *Burkholderia cenocepacia* in intrinsic antibiotic resistance. BMC Microbiol. 2009;9:200.
2. Cardona ST, Valvano MA. An expression vector containing a rhamnose-inducible promoter provides tightly regulated gene expression in *Burkholderia cenocepacia*. Plasmid. 2005;54(3):219-28.

Table S2. Primers used for PCR amplification. Restriction sites are underlined.

Primer	Sequence (5´ to 3´)	Annealing temperature	Primer length	Amplicon length (bp)
BCAL2462_F	TTTCATATGGAACCGCCACCC	64	21	581
BCAL2462_R	TTTAAGCTTCTAGATATCCAGTTCGTCGTAAAT		33	
BCAL1512-1510_F	TTTCATATGAAAACCTCCCCGTTGTCCGT	72	29	4,352
BCAL1512-1510_R	TTTAAGCTTTCAGTGCGCCGCCGATG		26	
BCAM1947-1945_F	TTTCATATGCTCTCCTTTTCCCTACGCAGG	72	30	5,904
BCAM1947-1945_R	TTTAAGCTTCTACTGCCCCGACCGCG		26	

Table S3. MIC of 11026103 against *B. cenocepacia* J2315 in the presence of transition metals. All metals were used as their respective chloride salts.

Metal ion	MIC of 11026103 (μg/ml)		
	**0.01 mM**	**0.1 mM**	**1 mM**
Mn^{II}	16	16	16
Co^{II}	16	16	16
Ni^{II}	16	16	16
Cu^{II}	16	16	16
Zn^{II}	16	16	16
no metal	16	16	16

Figure S1. The effect of BCAL2462 expression on the transcriptome of *B. cenocepacia* J2315. Expression changes were calculated in comparison to empty vector control experiment (J2315 + pSCrhaB2 with or without 0.01% rhamnose). Black borderline on symbols denotes induction with rhamnose.

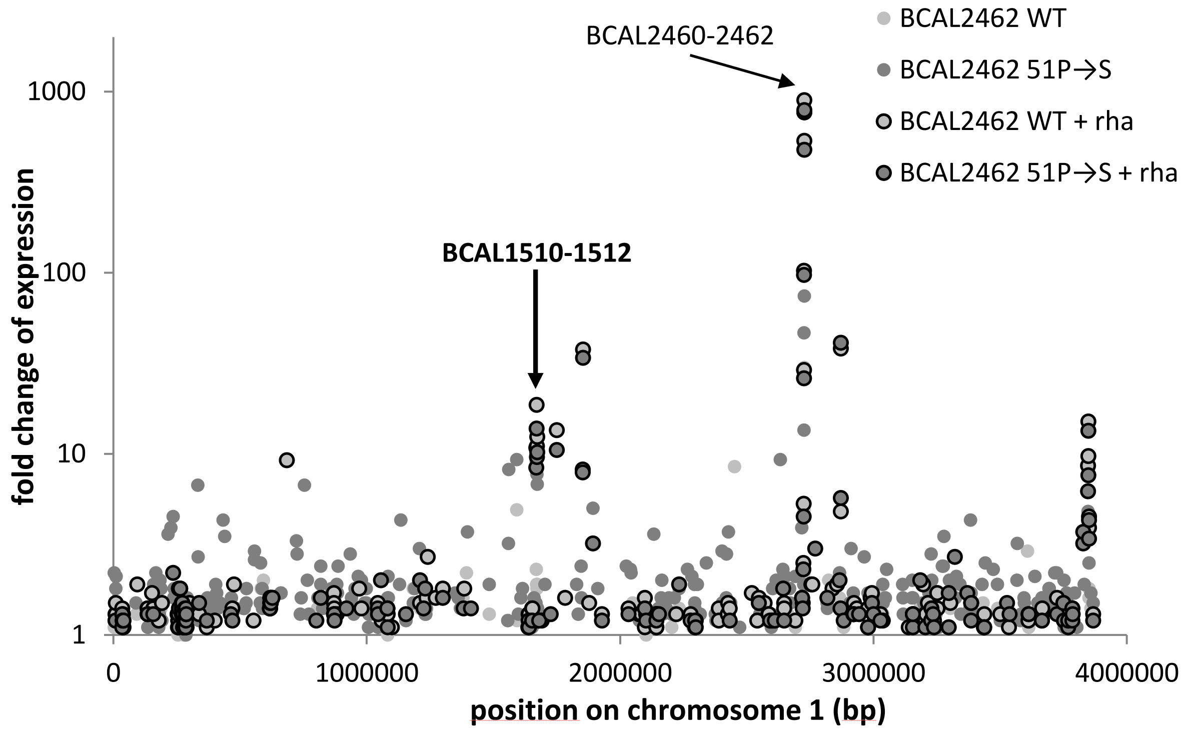

Figure S2. Comparison of chemical structure of 11026103 (this study) and HTP-2b (Salina *et al.*, Metallomics 2018). Structural differences are denoted in colored background.

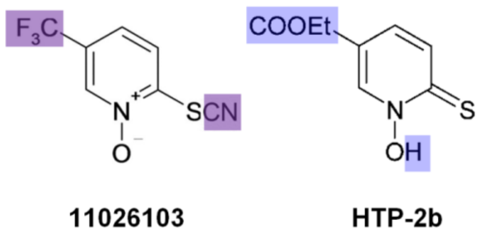

Figure S3. Concentration-dependent inhibitory effect of 11026103 on *B. cenocepacia* J2315. Mid-log phase cultures were treated with sub-inhibitory (5 µg/ml) and inhibitory (10 µg/ml and 15 µg/ml) concentrations of 11026103 (arrow) and viable count was determined by plating serial dilutions on LB agar. The values are average from three plate countings.

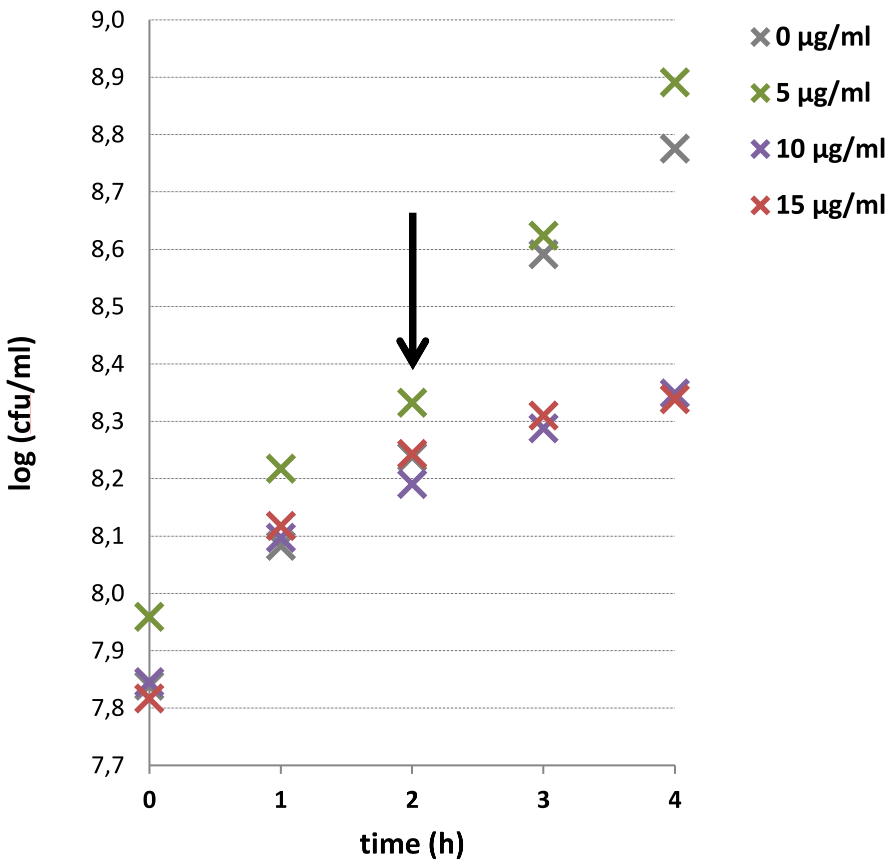

Figure S4. Effect of 11026103 on transcription of COG "Translation, ribosomal structure and biogenesis". The fold-change of expression is calculated with respect to untreated control experiment and is color-coded according to concentration of 11026103 used (sub-inhibitory: 5 µg/ml; inhibitory: 10 µg/ml and 15 µg/ml). Genes whose differential expression did not reach statistical significance (*p* – value greater than 0.2) were excluded.

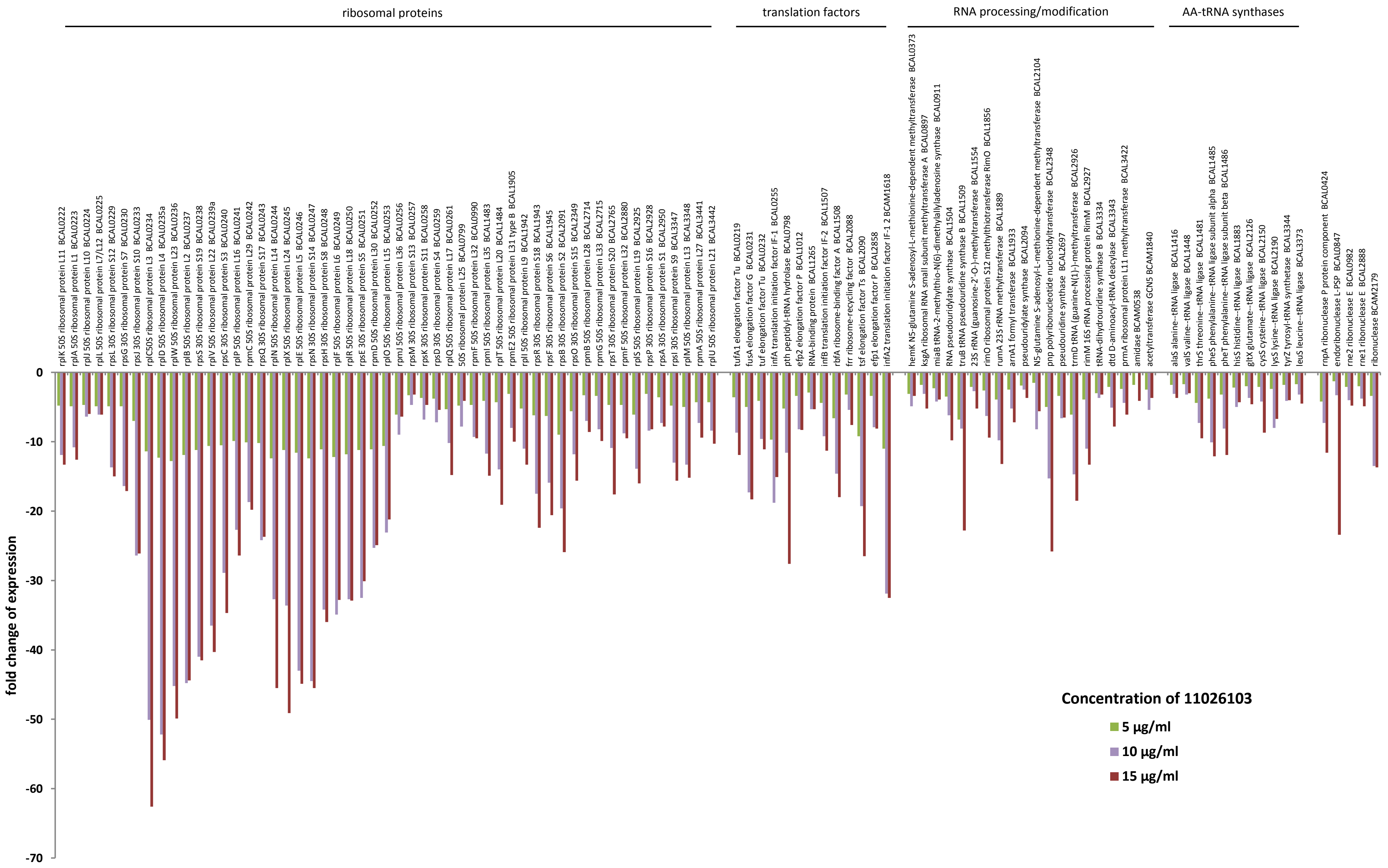

Supplement: Supplementary file 1 [file antibiotics-08-00159-s001.pdf]
